# Modeling Patient Journeys for Demand Segments in Chronic Care, With an Illustration to Type 2 Diabetes

**DOI:** 10.3389/fpubh.2020.00428

**Published:** 2020-08-28

**Authors:** Sylvia G. Elkhuizen, Jan M. H. Vissers, Mahdi Mahdavi, Joris J. van de Klundert

**Affiliations:** ^1^Erasmus School of Health Policy and Management, Erasmus University Rotterdam, Rotterdam, Netherlands; ^2^National Institute for Health Research/Tehran University of Medical Sciences, Tehran, Iran; ^3^Harvard T. H. Chan School of Public Health, The Bernard Lown Scholar for Cardiovascular Health, Department of Global Health and Population, Boston, MA, United States; ^4^Prince Mohammad bin Salman School for Business and Entrepreneurship/King Abdullah Economic City, King Abdullah Economic City, Saudi Arabia

**Keywords:** patient journey modeling, diabetes type 2, operations management, demand segmentation, generic framework

## Abstract

Chronic care is an important area for cost-effective and efficient health service delivery. Matching demand and services for chronic care is not easy as patients may have different needs in different stages of the disease. More insight is needed into the complete patient journey to do justice to the services required in each stage of the disease, to the different experiences of patients in each part of the journey, and to outcomes in each stage. With patient journey we refer to the “journey” of the patient along the services received within a demand segment of chronic care. We developed a generic framework for describing patient journeys and provider networks, based on an extension of the well-known model of Donabedian, to relate demand, services, resources, behavior, and outcomes. We also developed a generic operational model for the detailed modeling of services and resources, allowing for insight into costs. The generic operational model can be tailored to the specific characteristics of patient groups. We applied this modeling approach to type 2 diabetes (T2D) patients. Diabetes care is a form of chronic care for patients suffering diabetes mellitus. We studied the performance of T2D networks, using a descriptive model template. To identify and describe demand we made use of the following demand segments within the diabetes type 2 population: patients targeted for prevention; patients with stage 1 diabetes treated by their GP with lifestyle advice; patients with diabetes stage 2 treated by their GP with lifestyle advice and oral medication; patients with stage 3 diabetes treated by their GP with lifestyle advice, oral medication, and insulin injections; patients with stage 4 diabetes with complications (treated by internal medicine specialists). We used a Markov model to describe the transitions between the different health states. The model enables the patient journey through the health care system for cohorts of newly diagnosed T2D patients to be described, and to make a projection of the resource requirements of the different demand segments over the years. We illustrate our approach with a case study on a T2D care network in The Netherlands and reflect on the role of demand segmentation to analyse the case study results, with the objective of improving the T2D service delivery.

## Introduction

Demand for health services is increasing due to a growing and aging population, putting access, and healthcare expenditures under pressure (1). The increasing prevalence of chronic diseases in particular makes longer term health service delivery arrangements an important area for enhancing cost-effectiveness and efficiency. Insight into the resources correspondingly required is therefore a crucial prerequisite to tackle these challenges in delivering health care (2).

The variety of needs over different stages of chronic illness means that management and tailoring of health services is a complex task. This applies, for instance, to many patients with highly prevalent chronic conditions such as diabetes, COPD, and stroke. Modeling services to the needs of the “average patient” does not do justice to the journey of many patients and their individual requirements of services as their disease progresses. Moreover, models within the realm of the Chronic Care Model (3) often lack detail in process description and corresponding resource requirements which are key to determining service costs and effectiveness.

Detailed modeling of “patient pathways” for specific patient populations is another approach to these questions. However, patient pathways focus on addressing short term treatment episodes with clear beginnings and ends. They are especially suitable modeling for curable conditions and large and homogeneous patient groups (4). This, in turn, means they are less effective in addressing the journeys of patients who pass through various different stages in a chronic disease with its associated heterogeneity of demand.

The concept of “patient journey” has fairly recently emerged in literature as a method to analyse the sequential steps in the clinical process of the patient and improve its quality and safety (5). With “patient journey” we refer in this paper to the ‘journey’ of the patient along the services received within a demand segment of chronic care.

The purpose of this paper is to demonstrate that demand segmentation and an operations management approach can help to improve insight into the patient journey of patients with a complex chronic condition as diabetes type 2. The research questions addressed in this paper are: *How can we model the chronic condition patient journey in sufficient detail to be able to account for different needs, treatments, and outcomes relevant in different stages of the disease, and for different patient subpopulations? What are the results of applying demand segmentation to diabetes type 2 patients?*

Hence, our research objective is to develop new operational models which will explore the health service journeys of patients suffering from chronic conditions. The purpose is to advance insight into the relationship between operations (service delivery) on one hand, and outcomes and experiences at various relevant stages of the disease on the other. For stage of disease, therefore, we consider the set of patients currently in this stage as a subpopulation. We validate the developed model by conducting a case study in Type 2 Diabetes care in The Netherlands.

Our approach to modeling patient journeys is rooted in operations management. We consider the subpopulations as demand segments and, for each of these segments, needs are assumed to be homogenous. For each demand segment, the service operations to address the needs can be described using standardized service processes. This entails identifying the relevant process steps, and time relevant aspects and resources required for each of them. This operations management approach enables us to analyse how variation in processes relates to variation in health outcomes, costs, and experience, over the complete patient journey.

By way of background, Diabetes is defined as a chronic loss of capability to regulate the blood glucose level (6). A common distinction is between Type 1 Diabetes, where the body fails to produce insulin, and Type 2 Diabetes (T2D), where it is the way in which the body uses insulin which is important. Type 2 is the most common form of Diabetes. Its progression is characterized by an insidious onset and steady deterioration of the health state over a long period of time during which a complex of comorbid health conditions such as problems with heart, kidney, sight, and lower extremities may appear.

Treatment of T2D requires long term, continuous, and personalized care. Commonly, the larger part of health services for T2D, such as health promotion, health, education, diagnosis, regular monitoring, medication, check-up, is mostly performed by primary care professionals such as GPs and nurses (7). It is expected that the role of primary care professionals (as opposed to secondary care in hospitals and/or specialized physicians) will increase (8–10).

Due to the complexity and variety of T2D services, diabetes care typically involves multiple professionals to meet the demands of service users (11, 12). Consequently, providers usually establish relationships with other providers to integrate the elements of an often fragmented service process (13). This benefits different groups of stakeholders. As well as being useful to care providers, it is also of interest to service users, informal care givers, insurers, and policy makers. It may smooth the flow of service delivery by eliminating overlaps, delays, misuse, and overuse caused by fragmentation of service processes. Furthermore, it may help to contain costs (11).

Together, the collection of service providers involved forms a “health service provisioning” network. These networks can be formed by means of explicitly defined relationships, or more implicitly as collections of providers jointly visited by (a population of) T2D patients. The public health system arising from the National Health Service in the United Kingdom (UK) is a prime example of explicitly regionally organized provider networks with structural mechanisms for integration (14). In the Netherlands, a change in reimbursement schemes has also encouraged a variety of regional networks to be formed (15). These networks include GPs, dieticians, specialists, laboratory services, etc. (15). In other countries however, the networks may differ on a patient by patient basis as they choose the service providers to service their needs.

## Methods

### Operational Modeling

In Health Service Operations Management, operational models are used to describe and improve health service provisioning by networks of provider organizations. An operational model is a formal description of services that are performed to meet patient demands and that make use of structures to improve outcomes (16, 17). An operational model of a provider network, therefore, describes the components of operations of a network and the relationships between those components, thereby also enabling calculations of patient flows, resource use and costs to be made.

#### Framework

**Figure 1:**
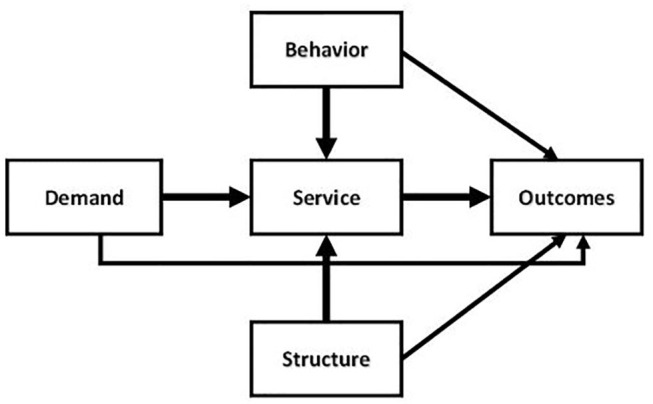
Generic health service operations framework.

As part of our methods, we present the generic framework developed for EU FP7 project Managed Outcomes (18, 19) which provided a comparative analysis of healthcare delivery networks for T2D, Ischaemic stroke, Osteo-arthritis patients, and Dementia in six EU countries (Finland, Germany, Greece, The Netherlands, Spain, UK). This generic framework, as presented in Figure 1, extends the well-known model of Donabedian (20).

The structure-process-outcome (SPO) model of Donabedian presents a seminal paradigm regarding the relationships between structure and process and patient outcomes. According to Donabedian, high quality structures are more likely to lead to high quality processes and, combined, they are more likely in turn to generate high quality outcomes (20). In this model, structure refers to factors such as (but not limited to) distribution and organization of human resources, financial resources, healthcare facilities, and ease of access by patients.

The main conceptual model of the relationships between operations and outcomes as depicted in Figure 1 contains not three but five entities, thereby extending Donabedian's Structure-Process-Outcome model. Based on this conceptual model the basic entities of provider networks are: *demand, behavior, structure, services*, and *outcomes*. The services replace Donabedian's processes, thus recognizing contemporary understanding of the nature of the co-creating interaction patterns between provider organizations and patients. The entity *structure* refers to the resources and other static features of the regional health service provider network operations. The structure defines the available tangibles and intangibles necessary to provide the services. Following Donabedian, a prime interest is to advance understanding of the impact of operations—in the form of services and the underlying *structures*—on *outcomes*. The entity *outcomes* refers to the results obtained through the service provisioning, for which we will distinguish health outcomes and service outcomes.

The new entity *demand* forms an extension of Donabedian's original SPO model and represents the demand for health services as resulting from the health conditions of individuals in the population. Demand may, by definition, form an independent determinant of outcomes and is therefore valuable in understanding outcomes and their relationships with operations. A second extension is the explicit inclusion of the entity *behavior*. The model takes into consideration both that health services may influence the patients' behavior, e.g., by inducing them to increase exercising, and also that patient *behavior* may affect the service provisioning. For instance, lack of therapy compliance by a patient may cause other, extra services to be required if their disease stage becomes altered as a result. By relating behavior to services, the model captures the common understanding that health service users are active co-creators of health services, rather than simply passive recipients (21). Correspondingly, the relation between behavior and services is thought to be bi-directional (22).

#### Operationalisation

The generic conceptual framework is further elaborated by disaggregating the five main entities into subcomponents which can subsequently be defined to analyse the journeys of a set of patient subpopulations through a provider network (see Table 1).

**Table 1:** Components and subcomponents of the generic health services network operations model.

**Component**	**Sub component**	**Definition**
Demand	Health service user	Service user refers to the individual patient who demands health services. Service user is defined with regard to demographic characteristics, disease history, and disease—specific medical conditions requiring the health services
	Demand segment	Segments refer to mutually exclusive subsets of the population of health service users with a common demand for health services (e.g., because of sharing a same health condition)
	Demand location	Locations define areas within the geographical areas which are meaningful to distinguish because of differences in demand and or geographical, socio-economic, and political characteristics
Service	Service element	A service element is the atomic unit of service For each service element the resource requirements specify the type of resources (see below) required to perform the service element, as well as the expected usage of each of these types (e.g., in hours) A service element can be described in terms of an operational performance (waiting times, frequency, length of stay, transitions to another service element) and a financial performance i.e., cost The costs of a service element are defined as the sum of the costs of the required resource usages (see below)
	Service journey	A service journey consists of a partially ordered set of service elements, which are provided to health service users from a demand segment Operational and financial performances of a service journey are aggregated from corresponding service elements performance The costs of a service journey are defined as the sum of the costs of the service elements involved Transition probability refers to the distribution of health service users from the demand segment corresponding to the service journey over possible succeeding demand segments (and corresponding service journeys)
	Service user journey	User journey refers to the sequence of services that a health service user follows (defined through the sequence of service journeys) The costs of a service user journey consist of the sum of the costs of the service journeys involved
Structure	Resource	A resource is a means to provide a service. Resources are described according to their type, availability, capacity and unit cost With regard to type, resources are distinguished into devices, facilities, and human resources Resource availability refers to the amount of resources which is available to deliver services per time period Resource capacity refers to the amount of health service users that can be treated in a time period Resource cost refers to the monetary cost of a resource per unit (e.g., per hour)
	Service provision point	Provision point refers to a location where resources required to provide a service are located Access to provision point is measured by physical distance of and travel time from the demand location of the health service user to the provision point
	Service provider	A health service provider is a person or a legal entity who/which delivers health services to patients.
Behavior	General health related behavior	General health behavior refers to the life style of the health service user, such as smoking, diet, and physical exercise behavior
	Service related behavior	Service related behavior refers to behavior which directly relates to the health services, e.g., treatment adherence or follow-up to advices by service provider
Outcome	Health outcomes	Health outcomes are features of the health care user's health. A variety of quite different health outcomes can be considered ranging from perceived health related quality of life as reported by the health service user to specific clinical outcomes as reported by the health care provider
	Service outcomes	Service outcomes regards both provider measures on service performance (such as waiting times) as well as health service users perceptions of service provisioning, and the valuation of the service provisioning by health service users

We now briefly discuss the level two components in Table 1. *Demand* describes health service users, in terms of, for example, their demographics and health conditions. The geography of demand can be described through demand locations, to which socio-economic characteristics (and others) can be attributed if desired to distinguish as confounder in the subsequent analysis. The population of health service users can be partitioned into segments (or subpopulations) for which different health services are provided. The atomic units by which *services* are defined are referred to as service elements (e.g., an outpatient visit). The next larger unit is the “service journey,” which is an ordered set of service elements describing the health service elements commonly used by a segment of health service users (e.g., according to an evidence-based clinical guideline). Over time service users may transition between different demand segments and, in doing so, they will follow different sequences of service journeys. Such a health service user's specific sequence of service journeys is referred to as a “service user journey.”

The *structures* underlying the service provisioning are partly defined in terms of current and non-current assets, such as buildings and equipment. Each of these resources has a type (e.g., X-ray machine) and an availability (e.g., weekdays 09.00 till 16.00), a capacity (e.g., 3 patients per hour) and a cost (e.g., €100 per hour). The resources are assigned to service providers (e.g., based on ownership) and located at service provision points. Service providers may have resources at various service provision points, and service provision points may hold resources from various service providers. Human resources also form part of the structure. Like the tangible resources they may have a type (e.g., general practitioner), availability (e.g., 32 h per week), capacity and cost.

The elaborated generic framework encompasses two types of *outcomes*, health outcomes, and service outcomes. Service outcomes refer to measurements and perceptions of the services provisioning by health service users, e.g., a service user's perceived timeliness or friendliness, or health service user satisfaction. Health outcomes may refer to generic health outcomes, such as patient reported quality of life, or to disease specific clinical outcomes such as HbA1c level (for T2D).

*Behavior* encompasses two kinds of behavior. Firstly it relates to generic health behavior, for instance referring to life style or diet. Secondly, it may refer to health service co-creating behavior, for instance to reporting measurements or therapy adherence (23).

#### Operational Model

**Figure 2:**
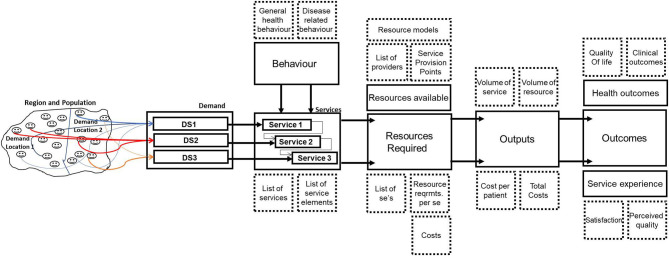
Generic operational model of patient journeys as used in managed outcomes.

Figure 2 illustrates how the elaboration of components and subcomponents can be used in an operational model to analyse the journeys of patients in different demand segments.

We see from Figure 2 that the demand from a population in a region can be disaggregated into demand locations and demand segments. A distinction in demand location might be, for instance, urban or rural—as patients living in a rural area typically have a further distance to travel, or might be living more healthily. A “demand segment” refers to a group of patients who have the same disease but are also comparable in the amount of resources required. For chronic diseases this requires often a distinction between stages of the disease, as further stages often require more—and different—resources than the early stages.

For each of the demand segments services can be defined, consisting of service elements and a journey along the service elements. Demand segments and patient journey per segment result in expected patient flows, taking also into account patient behavior impacts such as “no-show” at a clinical appointment. As the resource requirements are defined at the level of service elements, we can calculate the amount of resources required for each demand segment. Furthermore, we can calculate the annual output of the system under study, expressing this in the number of services produced and the number of resources required. We can then translate this into annual costs per patient in a demand segment (as the costs are defined at the level of service element).

The output of the system can be related to both health outcomes and service experience outcomes. When the same demand segments are used in all parts of the model, we can also differentiate outcomes between demand segments.

### Case Study

The operational models for diabetes, stroke, osteoarthritis, and dementia were developed in case studies performed in six countries as part of the EU project Managed Outcomes. For detailed information on the methodology used in this project see (18, 19).

As this paper is based on the case study performed for diabetes type 2 in The Netherlands, we will provide more information on the content of this case study. The case study took place in 2010–2011 in the region of The NieuwewaterwegNoord&DelflandWestlandOostland (NWN&DWO), a region covering 273 square kilometers to the Northwest of Rotterdam with 443.109 inhabitants. For the case study a project team was formed with a GP from the Primary Care Organization ZEL who was the coordinator of the program for diabetes, one of the managers in ZEL and two researchers from EUR. Based on the generic operational model (Figure 2) we developed together with the team a specific operational model for diabetes care in ZEL, using data on population and diabetes patients in the region and on the diabetes care delivered by GP practices as part of ZEL. As the case study in NL was in the lead for developing the operational model and templates for describing services and resources for the case studes in the other countries, we developed an intensive working relationship with our ZEL partners which spread over more than 1 year. The templates allowed other case studies to look at the operational model for ZEL and to change or add services and resources to allow for specific operational models elsewhere. The case study involved also data collection on the performance of diabetes care, such as HbA1c, and a survey among diabetes patients with questions on quality of life (EQ5D), service satisfaction, and experiences. The questionnaire for diabetes was developed together with our ZEL partners and tested in ZEL before being used in the case studies elsewhere. The results of the case studies in other countries were also shared with our partners in ZEL resulting in a paper on the diabetes project with our GP coordinator from ZEL as co-author (24).

## Results

This section presents a disease-specific model for a health service network to service the needs of a regional population of T2D patients. It uses data collected for a case study performed in The Netherlands as part of the Managed Outcomes project. First, in section Illustration of Specific Operational Model for Diabetes Patients, we outline the operational model, defining demand, services, structures behaviors, and outcomes. In section Differences in Resource Demand, Costs, and Outcomes we then study how patients journeys arise from the demand segments included in the model.

### Illustration of Specific Operational Model for Diabetes Patients

For Diabetes, different demand segments can be distinguished following the different stages of the disease (25):

DS1: Patients with high risk of developing diabetes
DS2: Patients with Diabetes type 2 treated with lifestyle advice
DS3: Patients with Diabetes type 2 treated with lifestyle advice and oral medication
DS4: Patients with Diabetes type 2 treated with lifestyle advice and insulin injections, sometimes also combined with oral medication
DS5: Patients with complicated type 2 diabetes treated by a physician specialized in diabetes care

**Figure 3:**
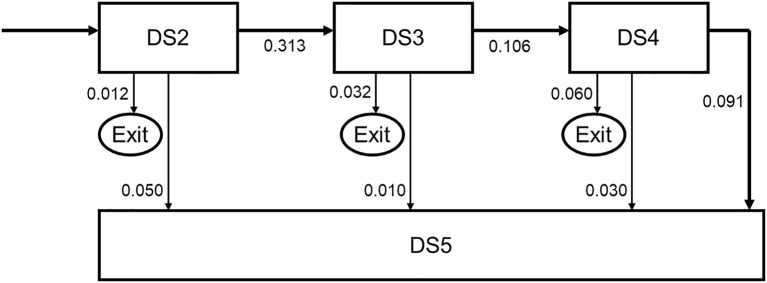
Movements between demand segments.

Patients may proceed from one demand segment to the next. From all segments, patients can move to the final Segment 5. Main movements between segments DS2-DS5 are shown in Figure 3. The transition probabilities to subsequent stages reflect the time per stage as well. For instance, a transition probability of 0.313 from stage DS2 to stage DS3, with an exit time probability of 0.012 and transition probability of 0.05 to stage DS5 imply a mean duration of DS2 of slightly <3 years.

For each demand segment, one or more health services (S) are defined (see Table 2).

**Table 2:** Services.

		**Delivered to demand segment**
S1	Screening	DS1
S2	Diagnosis	DS2
S3	Chronic diabetes treatment with lifestyle advice	DS2
S4	Chronic diabetes treatment with lifestyle advice and oral medication	DS3
S5	Chronic diabetes treatment with insulin therapy and lifestyle advice and/or oral medication, first year with insulin stabilization	DS4
S6	Chronic diabetes treatment with insulin therapy, lifestyle advice and oral medication, after first year	DS4
S7	Patients with complicated diabetes care treated with specialized care.	DS5

Each service is comprised of a list of service elements, which form the fundamental units of services delivered. For T2D, the list of service elements is depicted in Table 3, which also displays the resources required per service element, and the amount (in time or number) needed per resource per service element.

**Table 3:** Service elements and resources.

**Service element**		**Resource**	
**Number**	**Description**	**Description**	**Requirement**
SE1	Screening-visit	GP	20 min per unit
SE2	Lab test in GP office	Doctor assistant	5 min per unit
SE3	Lab-test-sampling	Lab	5 min per unit
SE4	Lab-test-analysis	Lab	1 min per unit
SE5	First visit	GP	20 min per unit
SE6	Visit for diagnosis and care plan	GP	20 min per unit
SE7	Follow–up visit	GP/Practice nurse	20 min per unit
SE8	Diet consultation	Dietician	45 min per unit
SE9	Eye care	Optometrist	5 min per unit
SE10	Foot care	Practice nurse	5 min per unit
SE11	Self–test glucose monitoring	test kit	1 kit per test
SE12	Oral medication	Medicine	1 pill per take
SE13	Insulin medication	Insulin	1 dose per injection
SE14	Education	Diabetic nurse	20 min per unit
SE15	Specialized care	Specialist	10 min per unit
SE16	Life style program	Life style consultant	20 min per unit
SE17	Insulin injection by professional	District nurse	5 min per unit
		Insulin	Dose per injection
SE18	Delivering medication by professional	Phamacist	5 min
SE19	Prescription medicine	GP	5 min
SE20	Education for using insulin	Diabetic nurse	20 min per unit

With the detailed resource use per service element specified, it is possible to relate the resources to the demand segments. By specifying the average percentage of patients actually using this service element and the frequency per user (see Table 4), the total resource requirement per service can be derived.

**Table 4:** Use of service elements per demand segment^*^ [frequency recommended per year, utilization rate (%)].

		**S1**	**S2**	**S3**	**S4**	**S5**	**S6**
SE1	Screening-visit	1					
SE2	Lab test in GP office						
SE3	Lab-test-sampling		1, 100%	1, 100%	1, 100%	1, 100%	1, 100%
SE4	Lab-test-analysis		1, 100%	1, 100%	1, 100%	1, 100%	1, 100%
SE5	First visit		1, 100%				
SE6	Visit for diagnosis and care plan		3, 100%				
SE7	Follow–up visit			4, 80%	4, 80%	4, 100%	4, 100%
SE8	Diet consultation			1, 10%	1, 1%	1, 1%	1, 1%
SE9	Eye care			1, 40%	1, 40%	1, 40%	1, 40%
SE10	Foot care			1, 10%	1, 65%	1, 65%	1, 65%
SE11	Self–test glucose monitoring					100, 100%	100, 100%
SE12	Oral medication				365, 100%	365, 90%	365, 90%
SE13	Insulin medication					365, 100%	365, 100%
SE14	Education		4, 100%				
SE15	Specialized care						
SE16	Life style program		12, 100%				
SE17	Insulin injection by professional					365, 2%	365, 2%
SE18	Delivering medication by professional				4, 100%	4, 100%	4, 100%
SE19	Prescription medicine				4, 20%		
SE20	Education for using insulin					8, 100%	

* *As this manuscript is focused on Type 2 Diabetes care at primary healthcare settings, demand segments 1-4 are further elaborated in terms of service elements, resources, behavior, and costs. We do not elaborate S7 for demand segment 5 since it was provided at hospital settings*.

### Differences in Resource Demand, Costs, and Outcomes

The service journeys and transitions between disease stages enables us to derive “expected service user journeys.” Moreover, they enable to derive the expected services requirements per stage, and corresponding activity-based costs.

Costs are derived using activity-based costing (26), where the service elements form the activities. “Cost objects” are service elements, services, service journeys, and ultimately service user journeys. “Cost drivers” include time, equipment, and medicine. For each service element required resource types are defined and, for each resource type, time is the default cost driver. Each resource type has a corresponding cost per time unit. The costs of each service element can therefore be calculated when the cost per time unit is specified for each resource type. For instance, a GP has a tariff per hour (or per minute), and the costs of a visit are the cost per minute times the number of minutes of the visit.

Cost calculations for the expected service journey S2 and S3 to service patients in demand segment 2 are illustrated in Table 5.

**Table 5:** Illustration of cost calculation for service journeys S2 and S3 serving demand segment 2.

			**S2**				**S3**		
		**Resource type**	Resource requirement per patient per year^*^	**Unit cost**	**Cost per patient per year**	**Cost in ZEL region per year (911 patients)**	Resource requirement per patient per year^*^	**Unit cost**	**Cost per patient per year**
SE3	Lab-test-sampling	Lab	5	€ 0.42	€ 2.10	€ 1,898	5	€ 0.42	€ 2.10
SE4	Lab-test-analysis	Lab	1	€ 0.42	€ 0.42	€ 380	1	€ 0.42	€ 0.42
SE5	First visit	GP	20	€ 1.10	€ 22.00	€ 20,043			
SE6	Visit for diagnosis and care plan	GP	60	€ 1.10	€ 66.00	€ 60,130			
SE7	Follow–up visit	GP&Nurse					64	€ 0.71	€ 45.44
SE8	Diet consultation	Dietician					5	€ 0.53	€ 2.65
SE9	Eye care	Optometrist					2	€ 1.10	€ 2.20
SE10	Foot care	Practice nurse					1	€ 0.29	€ 0.29
SE14	Education	Diabetic nurse	80	€ 0.58	€ 46.40	€ 42,516			
SE16	Life style program	Life style consultant	240	€ 0.58	€ 139.20	€ 127,549			
	Total per year				€ 276.12	€ 252,517			€ 53.10

* *Resource requirements are in minutes per year*.

Patients remain, on average, 3 years in demand segment 2, 1 year receiving service 2, and 2 years receiving service 3. The average cost per patient in DS2 is therefore 127,44 euros per year.

On a population level, the demand data as depicted in Table 6 enable to calculate the number of patients in each demand segment, based on incidence (the number of new T2D patients), and the transition probabilities between the demand segments as shown in Figure 3.

**Table 6:** Patients in case study region (2009).

	**Number/percentages**
Population	443.281
Number of new diabetes patients	910
Incidence	0,21%
Number of diabetes patients	12.218
Prevalence	2,76%

In combination with resource requirements per service journey, the operational model then facilitates to determine the expected total resource needs per demand segment and the corresponding expected total costs. Note that these cost calculations use the expected duration (in years) of patients stay in a demand segments (3 years in DS2, 9 years in DS3, and 10 years in DS4). Adding up the expected costs per segment yields the expected total cost of the service user journey (either per individual service user, or for the population at large).

For Type 2 Diabetes the most relevant outcome parameter to examine health status is the HbA1c level. Patients with HbA1c <53 mmol have balanced glucose levels. We also asked if the patient was satisfied with the services delivered (measured in a scale from 1–7); how they perceived their own health status (measured in a scale from 0 to 100); whether they were satisfied with their own health (measured in a scale from 1-7); and whether they knew their own HbA1c level.

In Table 7 we show the main results of these analyses^1^. As we focused in the case study on patients treated in primary care, the results are limited to the demand segments DS2-DS4.

**Table 7:** Main results per demand segment.

**Demand segment**	**Number of patients (2009) in segment**	**Hours of professional care per patient per year**	**Costs per patient per year**	**% Smokers**	**% Drinkers**	Satisfaction with services^*^ (1–7)	Own health^**^ (0–100)	Satisfaction with own health^**^ (1–7)	% Aware of HbA1c level^**^	**% Patients with HbA1c in control**
DS2	2687	3,1	127	7,0	72,0	5,9	77,1	4,9	42,4	84%
DS3	8084	1,5	419	11,4	66,8	6,3	77,8	5,0	56,1	87%
DS4	1451	2,6	1666	10,9	57,7	6,1	69,1	4,1	77,1	78%

* Significant p < 0.10;

** *significant p < 0.05*.

Patients in demand segment 2 (treatment with lifestyle advice) use the highest amount of professional care (3.1 h), but cost the least per year. Their patient journey includes, after the initial screening by the GP for the diagnosis, mainly lab tests and visits to the practice nurse for monitoring. However, in the first year there is much time invested in a lifestyle program, to try and keep the patient for as long as possible in this demand segment. If we consider the behavior of patients in DS2 we see the lowest level of smoking and the highest level of drinking. They are satisfied with the services delivered, but less so than in segments DS3 and DS4. They rate their health state as 77.1 on a scale 0–100 and are satisfied with their health, which is similar to DS3, but higher than DS4. The patients in DS2 are less aware of their HbA1c level. For 84% of the patients the HbA1c is in control, lower than for DS3, but higher than for DS4.

Patients in demand segment 3 (treatment with lifestyle and oral medication) use on average about 50% less professional care than patients in DS2, but their annual costs are about three times as much. Their patient journey differs from DS2 mainly in visits to the GP for medication prescription and the daily intake of oral medication. In terms of behavior, the percentage smokers is higher than for DS2 and the percentage drinkers lower. They are slightly more satisfied with the services compared with DS2. They rate their health state as 77.8, which is slightly higher than for DS2, and their satisfaction with health is similar than for DS2 but higher than for DS4. The patients in DS3 are more aware of their HbA1c level than for DS2. For 87% of the patients, slightly more than for DS2, the HbA1c is in control.

Patients in demand segment 4 (treatment with lifestyle, oral medication, and insulin injections) use on average slightly less professional care than in DS2, but their annual costs are about four times the costs of patients in DS3. The main differences in terms of patient journey with DS3 are in the initial education they receive for using insulin to do with daily self-medication and regular self-testing to monitor their glucose levels. In terms of behavior, the percentage of smokers is about similar to that of DS3 while the percentage of drinkers is lower. The patients in DS4 are slightly less satisfied with the services received. They rate their own health lower compared to patients in DS3. They are more aware of their HbA1c level, which is in control for 78% of these patients.

If we concentrate on the significance of the differences between demand segments, we see that the differences for smoking (*p* = 0.47) and drinking (*p* = 0.21) are not significant. The difference in satisfaction with services is moderately significant (*p* < 0.10). We see moderately significant differences between patients in demand segment 2 and 3 (*p* = 0.08).

There are significant differences in the perception of patients of their own health, and the satisfaction with their own health (*p* < 0.05). Patients in demand segment 4 feel less healthy compared with patients in demand segment 2 (*p* < 0.001) and patients in demand segment 3 (*p* = 0.001). Comparable differences can be seen in the satisfaction of patients with their own health. Patients in demand segment 4 are less satisfied compared with patients in demand segment 2 (*p* = 0.008) and patients in demand segment 3 (*p* = 0.001).

There are significant differences in the awareness of patients of their HbA1c level (*p* < 0.05). Patients in demand segment 2 are less likely to know their HbA1C level compared with patients in demand segment 3 (*p* = 0.061) and patients in demand segment 4 (*p* < 0.001). Patients in demand segment 3 also differ significantly with patients in demand segment 4 on this aspect (*p* = 0.019).

There are no significant differences between the demand segments concerning the percentage of patients within control for HbA1C.

## Conclusion and Discussion

In this paper we investigated how we can model the patient journey of patients with a chronic condition, and to address different needs, treatments, and outcomes, as relevant in different stages of the condition, and for different subpopulations with the condition. We analyzed the results of applying demand segmentation to diabetes type 2 patients.

The paper illustrates that patient journeys in chronic care and provider networks in a regional setting can be described, using standard operations management concepts and terminology. Distinguishing demand segments and using these demand segments throughout the different parts of the modeling and description of patient journeys and provider networks, makes it possible to look at the performance of regional health care delivery in transparent and verifiable detail.

The model enabled us to distinguish demand segments corresponding to stages of a chronic disease, and subsequently to model operations (services, resources, behaviors) and outcomes for each of these disease stages. The model also enabled recognition of differences in service provisioning for each stage, and in addition captured the transitions between disease stages. The results illustrate that the use of resources (and consequent activity-based costs) show significant differences for patients in different demand segments. We also observed some of the outcomes to differ significantly between patients belonging to different demand segments.

Therefore, an important benefit of this approach is that we can relate demand, services, resource use, costs, and outcomes at the more meaningful level of patient demand segments instead of only being able to consider the aggregate level of all diabetes type 2 patients. This more differentiated insight at demand segment level can be used to develop solutions for problems in the delivery of health services for type 2 diabetes patients.

Moreover, through modeling transitions between disease stages—and thus between demand segments and service journeys—we are able to model (expected) complete patient service journeys. For these complete service journeys, the model provides insight in overall performance, such as costs and quality of life over the full expected service user journey. This insight can help us to study the overall effects of interventions. For instance, one could study the effects of interventions aimed at keeping diabetes patients longer in DS2 with lifestyle advice, or increasing the role of nurses (24). The detailed underlying operational model facilitates to link interventions in patient journeys to outcomes, costs, and detailed resource requirements. This illustrates the added value of the operations management-based approach to demand segmentation followed in this paper.

One of the limitation of the paper is that the results of applying this modeling approach to diabetes type 2 are based on a case study and that the results will be different in other applications. Another limitation is that the insights on diabetes and diabetes care which have taken place since are not taken into account. The references used on diabetes and diabetes treatment may be outdated. Therefore, the contribution of the paper is mainly methodological. The methodology, however, can be used to develop new specific operational models based on up to date scientific insight into diabetes as complex chronic condition and treatment of diabetes, and—indeed—for other examples of chronic care as well.

## Data Availability Statement

The raw data supporting the conclusions of this article will be made available by the authors, without undue reservation.

## Ethics Statement

The studies involving human participants were reviewed and approved by Board of Directors of the Primary Care Group ZEL. Written informed consent for this study was not required in accordance with local legislation and national guidelines.

## Author Contributions

SE and JV prepared the draft text. MM and JK read the draft and provided comments for improvement. All authors were equally involved in the study.

## Conflict of Interest

The authors declare that the research was conducted in the absence of any commercial or financial relationships that could be construed as a potential conflict of interest.

## Footnotes

^1^Percentage smoker, drinkers, satisfaction with services, perception of own health, satisfaction with own health, % awareness of HbA1c, and percentage patients with HbA1c in control are based on questions in the survey; differences between demand segments were tested on significance with ANOVA.
